# Clinical and radiographic effects of ascorbic acid-augmented platelet-rich fibrin versus platelet-rich fibrin alone in intra-osseous defects of stage-III periodontitis patients: a randomized controlled clinical trial

**DOI:** 10.1007/s00784-021-03929-1

**Published:** 2021-04-12

**Authors:** Mohamed Talaat Elbehwashy, Manal Mohamed Hosny, Ahmed Elfana, Alaa Nawar, Karim Fawzy El-Sayed

**Affiliations:** 1 Oral Medicine and Periodontology Department, Faculty of Dentistry, Cairo University, Al Saraya Str. 11, Manial, Cairo, Egypt; 2 Oral and Maxillofacial Radiology Department, Faculty of Dentistry, Cairo University, Giza, Egypt; 3 Clinic for Conservative Dentistry and Periodontology, School of Dental Medicine, Christian Albrechts University, Kiel, Germany

**Keywords:** Vitamin C, Ascorbic acid, Platelet-rich fibrin, Periodontal regeneration, Periodontitis, Intra-osseous defects

## Abstract

**Aim:**

To assess platelet-rich fibrin (PRF) with ascorbic acid (AA) versus PRF in intra-osseous defects of stage-III periodontitis patients.

**Methodology:**

Twenty stage-III/grade C periodontitis patients, with ≥ 3 mm intra-osseous defects, were randomized into test (open flap debridement (OFD)+AA/PRF; *n* = 10) and control (OFD+PRF; *n* = 10). Clinical attachment level (CAL; primary outcome), probing pocket depth (PPD), gingival recession depth (RD), full-mouth bleeding scores (FMBS), full-mouth plaque scores (FMPS), radiographic linear defect depth (RLDD) and radiographic defect bone density (RDBD) (secondary-outcomes) were examined at baseline, 3 and 6 months post-surgically.

**Results:**

OFD+AA/PRF and OFD+PRF demonstrated significant intragroup CAL gain and PPD reduction at 3 and 6 months (*p* < 0.001). OFD+AA/PRF and OFD+PRF showed no differences regarding FMBS or FMPS (*p* > 0.05). OFD+AA/PRF demonstrated significant RD reduction of 0.90 ± 0.50 mm and 0.80 ± 0.71 mm at 3 and 6 months, while OFD+PRF showed RD reduction of 0.10 ± 0.77 mm at 3 months, with an RD-increase of 0.20 ± 0.82 mm at 6 months (*p* < 0.05). OFD+AA/PRF and OFD+PRF demonstrated significant RLDD reduction (2.29 ± 0.61 mm and 1.63 ± 0.46 mm; *p* < 0.05) and RDBD-increase (14.61 ± 5.39% and 12.58 ± 5.03%; *p* > 0.05). Stepwise linear regression analysis showed that baseline RLDD and FMBS at 6 months were significant predictors of CAL reduction (*p* < 0.001).

**Conclusions:**

OFD+PRF with/without AA significantly improved periodontal parameters 6 months post-surgically. Augmenting PRF with AA additionally enhanced gingival tissue gain and radiographic defect fill.

**Clinical relevance:**

PRF, with or without AA, could significantly improve periodontal parameters. Supplementing PRF with AA could additionally augment radiographic linear defect fill and reduce gingival recession depth.

**Supplementary Information:**

The online version contains supplementary material available at 10.1007/s00784-021-03929-1.

## Introduction

Periodontitis is an inflammatory destructive disorder of the periodontal supporting structures, associated with microbial dysbiosis [1]. A successful periodontal treatment aims to regenerate the lost periodontium to its original anatomy and function [2, 3]. Platelet-rich fibrin (PRF) is an easily prepared, autologous natural scaffold, harbouring a multitude of growth/differentiation factors, including platelet-derived growth factor (PDGF), transforming growth factor-β (TGF-β), insulin-like growth factor (IGF), vascular endothelial growth factor (VEGF) and fibroblast growth factor-β (FGF-β) [4–7], with a potential to promote periodontal repair/regeneration [8]. It has been investigated in combination with open flap debridement (OFD) and with a variety of biomolecules, including statins, metformin, bisphosphonates, and enamel matrix derivatives (EMD), to achieve a sustained release into periodontal defects [9].

Ascorbic acid (AA) is a potent antioxidant biomolecule, with a multitude of positive effects on oral and periodontal health [10, 11], on non-surgical periodontal therapy in smokers [12] and on periodontal disease prevention [11]. AA stimulates self-renewal and differentiation of periodontal stem/progenitor cells, boosts their telomerase activity [13], inhibits cellular senescence [14] and enhances pluripotency biomarker expression [15–17]. Yet, little evidence supports the clinical use of AA incorporated into PRF (AA/PRF) in management of intra-osseous periodontal defects.

To the best of our knowledge, this is the first randomized clinical trial conducted to evaluate clinical attachment level (primary outcome), probing pocket depth, gingival recession depth, full-mouth bleeding scores, full-mouth plaque scores, radiographic defect bone density and radiographic linear defect depth (secondary outcomes), following the application of AA/PRF versus PRF with OFD in intra-osseous defects of stage-III grade C periodontitis patients.

## Materials and methods

### Study design and registration

The study was designed as double-blind, parallel arms, randomized controlled clinical trial, with 1:1 allocation ratio to compare clinical and radiographic parameters of OFD with AA incorporated into PRF (OFD+AA/PRF; test group) versus PRF alone (OFD+PRF; control group) in intra-osseous defects’ therapy. The research protocol was registered on www.clinicaltrials.gov on October 2018 (NCT03707483). Research protocol and informed consent templates were approved by the Ethics Committee, Faculty of Dentistry, Cairo University on December 2018 (IRB:18|12|13). The study was carried out and reported in compliance with the EQUATOR guidelines and ethical principles of the Helsinki Declaration for medical research involving human subjects as revised in Fortaleza 2013.

### Participants

Recruiting potential participants was carried out through screening of patients admitted to the Department of Oral Medicine and Periodontology at the Faculty of Dentistry, Cairo University, Egypt, personal referral and poster announcements, until achieving the targeted sample size adjusted for possible dropouts. Participants were screened, operated and followed up from March 2019 till June 2020. All participants were stage-III grade C periodontitis patients with mandibular molar teeth showing no mobility nor furcation involvement, with ≥ 5 mm clinical attachment loss and ≥ 3 mm two- or three-walled intra-osseous defects of as detected radiographically. For grading, the percentage of bone loss was used at the worst affected tooth in the dentition divided by the patient’s age [18]. Only motivated adult participants (age ≥ 18), presented with full-mouth bleeding or plaque scores ≤ 20% at the time of surgical interventions [19], were included. Smokers, diabetic patients, patients with systemic conditions contradicting surgical intervention and pregnant or nursing women were excluded (Fig. S1).

### Sample size

Sample size was calculated using a mean CAL difference of 1 mm as the minimum clinically acceptable value and a standard deviation of 0.68 mm [20]. Using *β* = 80% and *α* = 5%, and based on independent *t*-test, 8 defects were deemed necessary in each group (PS 3.1.2, Vanderbilt University, Tennessee, USA). This number was increased to 10 defects/group to compensate for 20% anticipated dropouts during follow-up.

### Randomization

Intra-osseous defects were randomly assigned to be treated using OFD+AA/PRF or OFD+PRF with a 1:1 allocation ratio. Sequence generation and concealment were carried out by a single investigator (MH), using www.random.org. Allocation was concealed in serially numbered, identical and opaque sealed envelopes. KFE was responsible for assigning participants to the corresponding group. All participants were enrolled and equally prepared for the surgical procedure by a single investigator (ME). Following OFD, the allocation was revealed (KFE) to the operator (ME) according to the sequence.

### Blinding

Study participants were blinded. The operator couldn’t be blinded. Outcomes’ assessors and biostatistician were blinded. Participants’ identity and their corresponding study group were masked by assigning an identification number to data files for data transfer to and from assessors.

### Outcomes

Clinical attachment level (CAL, primary outcome) from cementoenamel junction (CEJ) to base of pocket, gingival recession depth (RD) from CEJ to gingival margin and probing pocket depth (PPD) from gingival margin to base of pocket [21] were measured at baseline, 3 and 6 months for six sites per tooth, and the highest value was chosen for analysis [22]. Full-mouth bleeding score (FMBS) [23] and full-mouth plaque score (FMPS) [24] were measured at baseline and 6 months post-surgically. Measurements were taken using a Williams graduated periodontal probe (Martin™ periodontal probe No. 43-357-00, KLS Martin, Tuttlingen, Germany). Changes were calculated by subtracting 3 and 6 months from baseline values, and percentage changes were obtained by dividing change values by baseline numbers.

**Fig. 1:**
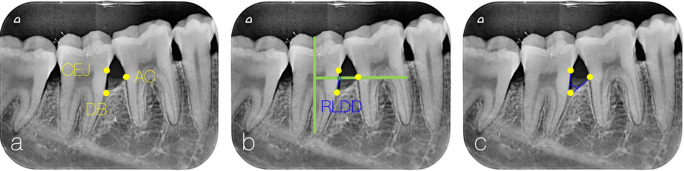
Intra-osseous defect radiographic measurements. **a** Identifying reference points: cementoenamel junction (CEJ), defect base (DB) and alveolar crest (AC). **b** Identifying reference lines (in green): vertical line corresponding to long axis and horizontal perpendicular line passing through AC, and identifying radiographic linear defect depth (RLDD) in blue. **c** Radiographic angle connecting CEJ, DB and the lateral border of the defect

Radiographic linear defect depth (RLDD; secondary outcome) was measured as the depth of intra-osseous defect from alveolar crest (AC) to defect base (DB) at baseline and 6 months. Customized bite blocks were fabricated for each site, using diagnostic casts and acrylic resin. Using XCP X-ray Holder kit (Dentsply Sirona, Charlotte, USA) and PSP sensor size two (Xios AE, Dentsply Sirona), parallel-angel standardized radiographs were obtained (60 kVp, 8 mA, and 0.10 s, Heliodent Plus, Dentsply Sirona). Using ImageJ software (Research Services Branch, NIH, Bethesda, Maryland, USA), three reference points relevant to each defect site, namely, CEJ, AC and DB, and three reference lines, the long axis of the concerned tooth, a line parallel to the root surface from CEJ to DB, and another starting from AC ending perpendicularly on the long axis line, were identified. Radiographic linear defect depth (RLDD) was measured over the DB-CEJ line on baseline and follow-up radiographs as the distance from DB to the intersection point with the line between AC and the long axis line [25]. Defect angle at baseline was determined as the angle between lines connecting CEJ to DB and lateral defect border [26] (Fig. 1).

For radiographic defect bone density (RDBD; secondary outcome) assessment, the region of interest (ROI) was outlined, through drawing an outline corresponding to the demarcating walls of the intra-osseous defect, and mean grey values were calculated. ROIs were not superimposed on any portion of the tooth surface. The measured area customized for each baseline radiograph was duplicated on the 6-month radiographs, and grey value changes were calculated [27].

### Calibration

Outcomes were recorded by two blinded calibrated investigators, one for clinical parameters (AE) and the other for radiographic measures (AN). Calibration was done before study conduction by measuring relevant data (not included in the study) twice 1 week apart. All measurements were repeated, retrieving intra-examiner agreement scores of 0.85 for CAL, PPD and RD and 0.82 for radiographic measurements.

### Preoperative phase

Participants meeting all inclusion criteria proceeded to radiographic examination were provided with information about the study, undersigned an informed consent, received phase-I periodontal therapy of supra- and subgingival debridement and were instructed to maintain proper oral hygiene by teeth brushing and daily use of 0.12% chlorhexidine HCL mouthwash (Hexitol, ADCO Pharma Co, Cairo, Egypt) [28]. After 4–6 weeks, re-evaluation was performed to confirm the need for periodontal surgery (persistence of interproximal defect with PPD ≥ 5 mm, clinical attachment loss ≥ 5 mm and radiographic intra-osseous defect ≥ 3 mm) [29].

### Surgical phase

All surgeries were conducted by a single operator (ME). On the day of surgery (baseline) FMPS, FMBS, CAL, PPD and RD were recorded and standardized periapical radiographs were taken. A full-thickness mucoperiosteal flap was raised, around the affected tooth and one adjacent tooth mesial and distal using 15C surgical blades (KLS Martin GmbH, Tuttlingen, Germany). Thorough OFD the defects were instrumented, using mini-five and after-five Gracey curettes (Hu-Friedy, Chicago, USA) under local anesthesia (2% mepivacaine HCl with 1:20000 levonordefrin, Alexandria Co. for Pharmaceuticals, Egypt).

**Fig. 2:**
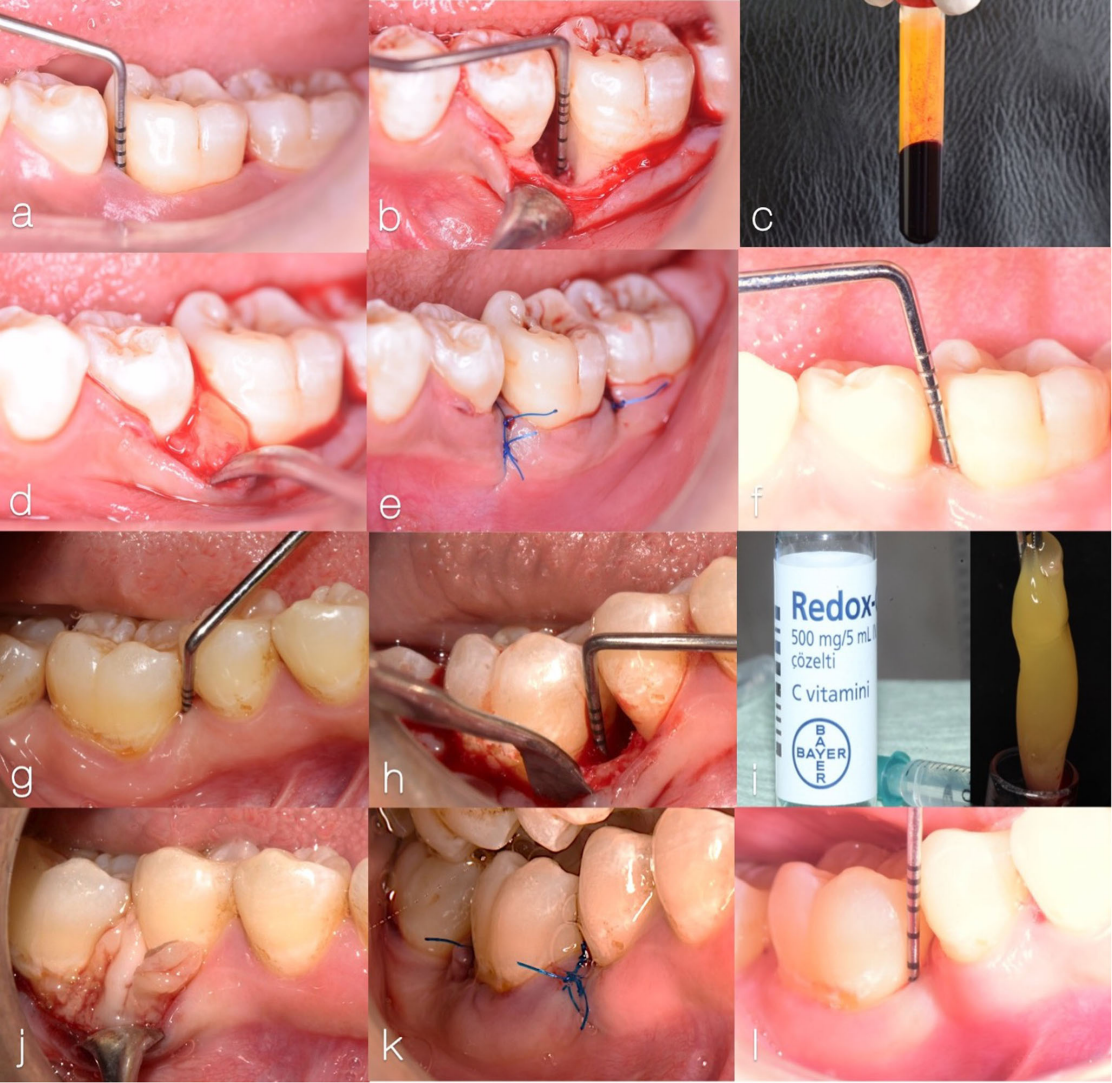
Clinical steps in representative cases of the control group, using open flap debridement (OFD) and platelet-rich fibrin (PRF) (**a**–**f**) and test group using OFD and AA/PRF combination [81]: **a** 7-mm probing pocket depth mesial to lower left six at baseline. **b** Intra-osseous defect with vertical component of 5 mm. **c** Preparation of PRF. **d** Application of PRF plug into the periodontal defect. **e** Internal vertical mattress sutures. **f** 3-mm probing pocket depth after 6 months. **g** 8-mm probing pocket depth mesial to lower right six at baseline. **h** Intra-osseous defect with vertical component of 5 mm. **i** Pure AA vial (left) and AA/PRF (right). **j** Application of the AA/PRF plug into the periodontal defect. **k** Internal vertical mattress suture. **l** 2-mm probing pocket depth after 6 months

For PRF preparation, 10 mm of fresh blood was drawn by venipuncture of the antecubital vein and collected into a blood collection tube without anticoagulant. In the AA/OFD+PRF group, 2500 μg pure AA (Redox-C, BAYER, Istanbul, Turkey) was added to the fresh blood to achieve a concentration of 250 μg/ml AA [15–17], and the tube was centrifuged at 400 g (3000 rpm) for 10 min at room temperature as described before [30]. The resultant PRF clot was compressed between two sterile gauze pieces and placed into the intra-osseous defect [31]. Flaps were repositioned and secured with 5-0 internal vertical mattress sutures (ASSUT, Pully-Lausanne, Switzerland, Fig. 2).

### Postoperative care and follow-up

1g E-moxclav (875 mg amoxicillin + 125 mg clavulanic acid, EIPICO, Tenth of Ramadan, Egypt) was administrated orally twice/day for 6 days. Participants were instructed to avoid hard brushing or trauma to the surgical site for 4 weeks, during which plaque was controlled by prescribing 0.12% chlorhexidine HCL oral rinse (Hexitol, ADCO Pharma Co, Cairo, Egypt) twice daily for 1 min [32]. Sutures were removed 14 days following surgery. Participants were instructed to continue tooth cleaning, using an ultra-soft toothbrush and roll technique for 1 month, and then resume normal brushing using a soft toothbrush. Professional plaque control was done monthly for 6 months [33].

### Statistical analysis

Data was explored for normality, using Kolmogorov-Smirnov and Shapiro-Wilk tests. Chi-square and Fisher’s exact tests were used for categorical baseline data. Numerical data were presented as means and standard deviation (SD). Both treatment groups were compared, using an independent Student’s *t*-test. Mean difference and 95% confidence interval (95% CI) were stated for each comparison. For intragroup comparisons, repeated measure ANOVA with Bonferroni correction was used for RLDD, FMBS and FMPS, a paired *t*-test was used. Additionally, a stepwise linear regression model was constructed, using CAL gain after 6 months as the dependent variable, while study group, age, gender, number of defect walls, FMBS at baseline, FMBS at 6 months, FMPS at baseline, FMPS at 6 months, radiographic angle and RLDD at baseline were the independent variables. All tests were two-tailed and *p* < 0.05 was considered statistically significant (SPSS for Windows, version 26, IBM, New York, USA).

## Results

### Baseline characteristics

This randomized, parallel-group clinical trial included a total of 20 intra-osseous defects in 20 participants (3 males and 17 females) diagnosed with stage-III grade C periodontitis. The OFD+PRF group included 1 male and 9 females with a mean (± SD) age of 28.2 ± 5.63 years, while the OFD+AA/PRF group included 2 males and 8 females (32 ± 5.27 years). The study was completed with no loss to follow-up. No unexpected adverse events were noticed clinically or reported by participants, such as allergy, abscess or surgical site exposure, and the healing processes were uneventful. Distribution of age and gender was balanced between test and control (*p* > 0.05). OFD+AA/PRF group contained 50% combined one-two-wall, 40% two-wall and 10% combined two-three-wall defects, while OFD+PRF group comprised 60% two-wall, 30% three-wall and 10% combined two-three-wall defects (*p* = 0.038, Chi-square). Baseline defect angle showed means of 44.68° ± 4.39° and 36.69° ± 6.22° in OFD+AA/PRF and OFD+PRF, respectively (*p* = 0.004, *t*-test, Table 1).

**Table 1:** Participant’s baseline parameters and distribution and configuration of intra-osseous defects (significant differences are marked with asterisk; *p < 0.05)

	OFD+AA/PRF *n* = 10	OFD+PRF *n* = 10	*p* Value
Age in years [mean (SD)]	32 (± 5.27)	28.2 (± 5.63)	0.137
Gender			
Male [*n* (%)]	2 (20%)	1 (10%)	1.00
Female [*n* (%)]	8 (80%)	9 (90%)	
Distribution of teeth with defects			
Anterior [*n* (%)]	5 (50%)	2 (20%)	0.371
Premolar [*n* (%)]	2 (20%)	3 (30%)	
Posterior [*n* (%)]	3 (30%)	5 (50%)	
Morphology of intra-osseous defects			
Combined 1–2 walls [*n* (%)]	5 (50%)	0 (0%)	
2 walls [*n* (%)]	4 (40%)	6 (60%)	0.038*
Combined 2–3 walls [*n* (%)]	1 (10%)	1 (10%)	
3 walls [*n* (%)]	0 (0%)	3 (30%)	
Intra-osseous defect angle	44.68° (± 4.39)	36.69 (± 6.22)	0.004*

### Clinical attachment level (CAL)

Statistically significant CAL gain was notable in both groups over time (*p* < 0.001, ANOVA/Bonferroni). OFD+AA/PRF showed CAL gain of 4.20 ± 1.18 mm (51.13 ± 12.70%) and 4.25 ± 1.27 mm (51.93 ± 13.91%), while OFD+PRF demonstrated CAL gain of 4.05 ± 086 mm (55.06 ± 14.04%) and 3.90 ± 1.15 mm (52.82 ± 16.07%) at 3 and 6 months respectively, with no significant differences observed between the groups at 3 and 6 months (*p* ≥ 0.05, independent *t*-test, Table 2).

**Table 2:** Mean (± SD), mean difference [95% CI] for clinical attachment level (CAL), probing pocket depth (PPD), recession depth (RD), full-mouth bleeding scores (FMBS) and full-mouth plaque scores (FMPS) (significant differences are marked with asterisk; *p < 0.05, CI: confidence interval)

	OFD+AA/PRF Mean (± SD) *n* = 10	OFD+PRF Mean (± SD) *n* = 10	Mean difference [95% CI]	Intergroup *p* value
Clinical attachment level (CAL)				
Baseline (mm)	8.25(± 1.50)	7.45 (± 0.86)	0.80 [− 0.35, 1.95]	0.160
At 3 months (mm)	4.05 (± 1.34)	3.40 (± 1.20)	0.65 [− 0.55, 1.85]	0.268
At 6 months (mm)	4.00 (± 1.55)	3.55 (± 1.32)	0.45 [− 0.90, 1.90]	0.493
Intragroup *p* value	< 0.001*	< 0.001*		
Gain at 3 months (mm)	4.20 (± 1.18)	4.05 (± 0.86)	0.15 [− 0.82, 1.12]	0.750
Gain at 3 months (%)	51.13 (± 12.70)	55.06 (± 14.04)	− 3.93 [− 16.51, 8.65]	0.520
Gain at 6 months (mm)	4.25 (± 1.27)	3.90 (± 1.15)	0.35 [− 0.79, 1.49]	0.527
Gain at 6 months (%)	51.93 (± 13.91)	52.82 (± 16.07)	− 0.89 [− 14.67, 12.88]	0.893
Probing pocket depth (PPD)				
Baseline (mm)	6.80 (± 1.36)	6.90 (± 0.97)	− 0.10 [− 1.21, 1.01]	0 .852
At 3 months (mm)	3.50 (± 1.18)	2.95 (± 0.86)	0.55 [− 0.43,1.53]	0.250
At 6 months (mm)	3.35 (± 1.36)	2.80 (± 0.86)	0.55 [− 0.35, 1.45]	0.217
Intragroup *p* value	< 0.001*	< 0.001*		
Reduction at 3 months (mm)	3.30 (± 1.36)	3.95 (± 0.60)	− 0.65 [− 1.64, 0.34]	0.183
Reduction at 3 months (%)	47.86 (± 16.77)	57.77 (± 9.02)	− 9.91 [− 22.57, 2.74]	0.117
Reduction at 6 months (mm)	3.45 (± 1.36)	4.10 (± 0.61)	− 0.65 [− 1.64, 0.34]	0.186
Reduction at 6 months (%)	49.91 (± 14.37)	59.91 (± 8.89)	− 10.00 [− 21.23, 1.22]	0.077
Recession depth (RD)				
Baseline (mm)	1.45 (± 0.76)	0.55 (± 0.55)	0.90 [0.28, 1.52]	0.007*
At 3 months (mm)	0.55 (± 0.69)	0.45 (± 0.64)	0.10 [− 0.52, 0.72]	0.740
At 6 months (mm)	0.65 (± 0.82)	0.75 (± 0.68)	− 0.10 [− 0.81, 0.61]	0.769
Intragroup *p* value	0.003*	0.361		
Reduction at 3 months (mm)	0.90 (± 0.74)	0.10 (± 0.77)	0.80 [− 0.09, 1.51]	0.029*
Reduction at 6 months (mm)	0.80 (± 0.71)	− 0.20 (± 0.82)	1.00 [0.28, 1.72]	0.010*
Full-mouth bleeding score (FMBS)				
Baseline	11.01 (± 3.25)	10.27 (± 2.46)	0.74 [− 1.96, 3.45]	0.571
6 months	10.78 (± 2.21)	11.18 (± 2.54)	− 0.40 [− 2.63, 1.84]	0.715
Intragroup *p* value	0.880	0.333		
Full-mouth plaque score (FMPS)				
Baseline	11.72 (± 2.59)	10.98 (± 2.77)	0.74 [− 1.78, 3.26]	0.546
6 months	12.63 (± 2.46)	13.20 (± 2.47)	− 0.57 [− 2.88, 1.75]	0.614
Intragroup *p* value	0.456	0.124		

### Probing pocket depth (PPD)

OFD+AA/PRF showed a significant PPD reduction of 3.30 ± 1.36 mm (47.86 ± 16.77%) and 3.45 ± 1.36 mm (49.91 ± 14.37%), while OFD+PRF demonstrated a significant PPD reduction of 3.95 ± 0.60 mm (57.77 ± 9.02%) and 4.10 ± 0.61 mm (59.91 ± 8.89%) at 3 and 6 months, respectively, with no significant difference observed between the groups at 3 and 6 months (*p* ≥ 0.05, independent *t*-test, Table 2).

### Recession depth (RD)

In OFD+AA/PRF, a significant RD reduction of 0.90 ± 0.74 mm and 0.80 ± 0.74 mm was notable at 3 and 6 months, respectively (*p* = 0.003), while OFD+PRF demonstrated a RD reduction of 0.10 + 0.77 mm at 3 months with a rebound RD-increase of 0.20 ± 0.82 mm at 6 months (*p* = 0.361, ANOVA/Bonferroni). Significant RD reduction in favour of OFD+AA/PRF was notable at 3 and 6 months (*p* = 0.029 and *p* = 0.010, respectively, independent *t*-test, Table 2).

### Full-mouth bleeding (FMBS) and plaque scores (FMPS)

In OFD+AA/PRF, FMBS was 11.01 ± 3.25% and 10.78 ± 2.21%, versus 10.27 ± 2.46% and 11.18 ± 2.54% in OFD+PRF at baseline and 6 months, respectively. For FMPS, OFD+AA/PRF showed 11.72 ± 2.59% and 12.36 ± 2.46%, versus OFD+PRF, which demonstrated 10.89 ± 2.77% and 13.20 ± 2.47% at baseline and 6 months, respectively, without significant differences either within (*p* ≥ 0.05, paired *t*-test) or between groups (*p* ≥ 0.05, independent *t*-test, Table 2).

### Radiographic linear defect depth (RLDD) and radiographic defect bone density (RDBD)

Both OFD+AA/PRF and OFD+PRF demonstrated a statistically significant RLDD reduction at 6 months compared to baseline (*p* ≤ 0.001, paired *t*-test). A mean RLDD reduction of 2.29 ± 0.61 mm (48.30 ± 8.30%) was demonstrated in OFD+AA/PRF, while OFD+PRF showed 1.63 ± 0.46 mm (40.62 ± 9.59%) reduction at 6 months (*p* = 0.014). OFD+AA/PRF and OFD+PRF exhibited an increase in RDBD of 14.61 ± 5.39% and 12.58 ± 5.03% at 6 months (*p* = 0.395, independent *t*-test, Table 3).

**Table 3:** Changes in mean (± SD) for radiographic linear defect depth (RLDD) and radiographic defect bone density (RDBD)

	OFD+AA/PRF Mean (± SD)	OFD+PRF Mean (± SD)	Mean difference [95% CI]	Intergroup *p* value
Defect depth (RLDD)				
At baseline (mm)	4.69 (± 0.76)	3.98 (± 0.43)	0.71 [0.12, 1.30]	0.022*
At 6 months (mm)	2.40 (± 0.45)	2.35 (± 0.42)	0.05 [− 0.36, 0.46]	0.817
Intragroup *p* value	< 0.001*	< 0.001*		
Defect depth reduction after 6 months (mm)	2.29 (± 0.61)	1.63 (± 0.46)	0.66 [0.15, 1.17]	0.014*
Defect depth reduction after 6 months (%)	48.30 (± 8.30)	40.62 (± 9.59)	7.68 [− 7.48, 16.10]	0.072
Radiographic defect bone density (RDBD) increase (%)	14.61 (± 5.39)	12.58 (± 5.03)	− 2.03 [− 6.93, 2.87]	0.395

Significant differences are marked with asterisk; *p < 0.05, CI: confidence interval

### Stepwise linear regression analysis

Stepwise linear regression analysis showed a direct correlation between RLDD at baseline and CAL gain (*p* < 0.001) as well as an inverse correlation between FMBS at 6 months and CAL gain (*p* = 0.023, Table 4).

**Table 4:** Significant predictors of CAL gain according to stepwise linear regression analysis model

Variables	β	SE	95% CI		*p* Value
			Lower limit	Upper limit	
Age	− 0.005	0.046	− 0.110	0.099	0.912
Gender	0.261	0.578	− 1.046	1.568	0.662
Treatment	− 0.565	0.604	− 1.932	0.802	0.374
Number of walls in the defect	0.261	0.275	− 0.363	0.884	0.369
FMBS at baseline	− 0.016	0.079	− 0.195	0.163	0.841
FMBS after 6 months	− 0.193	0.078	− 0.357	− 0.029	0.023*
FMPS at baseline	− 0.100	0.092	− 0.308	0.108	0.305
FMPS at 6 months	− 0.108	0.118	− 0.375	0.158	0.383
RLDD at baseline	1.395	0.256	0.854	1.936	<0.001*
Radiographic defect angle	− 0.026	0.041	− 0.120	0.067	0.538

*β* regression coefficient, *SE* standard error, *CI* confidence interval, *FMBS* full-mouth bleeding score, *FMPS* full-mouth plaque score, *RLDD* radiographic linear defect depth, significant differences are marked with asterisk; *p < 0.05

## Discussion

Periodontitis is a chronic multifactorial inflammatory disease, affecting the teeth supporting structures [1], causing alveolar bone destruction with horizontal and vertical bony defects. These intra-osseous defects are often associated with deep residual pockets, worsening the teeth long-term prognosis [29, 34, 35]. Periodontal therapy of intra-osseous defects aims to restore the lost periodontal structures, prevent the progression of periodontal destruction and enhance the tooth prognosis [36]. In the present study, the effect of AA augmented PRF was clinically investigated for the first time in the OFD of periodontitis-induced intra-osseous defects.

OFD remains to be one of the evidence-based periodontal surgical techniques [37, 38] for surgical therapy of intra-osseous defects [29] with remarkable results [39, 40]. Sites included in the current study presented with ≥ 5 mm clinical attachment loss and ≥ 3 mm three or two walls, or combined intra-osseous defects [41, 42]. Having almost identical molecular weights, the incorporation of AA into the PRF plugs relied on a previously reported method for metronidazole inclusion into PRF [30]. The employed PRF spin protocol was comparable to previous studies, exploring the effect of PRF combined with a variety of biological agents [26, 43–47] and using the same spin protocol. A split-mouth design was avoided to exclude any systemic effects of the applied AA on the control group, through the individual’s circulation. Comparable to earlier clinical trials on PRF, a 6-month follow-up period was selected [26, 48–54]. Smokers were excluded to avoid the retarding effect of smoking on periodontal wound healing [55].

Four prerequisites are pivotal to achieve periodontal repair/regeneration, namely, the cells, the adequate blood supply, the suitable scaffold directing the repair/regeneration process and finally the signaling biomolecules, modulating the cellular activities [56–58]. Aside from the physical properties of the defect-filling PRF haemostatic plug, platelets stimulate the proliferation and activation of a variety of cells involved in the repair/regeneration process, in addition to the release of a variety of growth, adhesion, coagulation and angiogenic factors into the defect site [59]. Through its fibrin content, the PRF plug could further provide a three-dimensional structural framework for regenerating periodontal cells [60].

In line with the current investigation, previous randomized controlled trials demonstrated the efficacy of PRF with OFD in achieving remarkable periodontal repair of intra-osseous defects [47, 61–64]. PRF with OFD could improve CAL, PPD and radiographic defect fill comparable or even superior to OFD in combination with bone grafts [65]. An incorporation of regenerative biomolecules into the PRF could further augment these effects [9]. Thus, PRF has been utilized as an autologous carrier for local delivery EMD, growth and morphogenetic/angiogenic factors, antibiotics and anti-osteoporotic molecules [26, 30, 66]. Through its degradation process, PRF could provide a gradual release of the incorporated biomolecules over 10–14 days [66], with superior results in the treatment of periodontal intra-osseous defects [67, 68]. At 250 μg/ml, AA was noted to maximally stimulate the proliferation, pluripotency and differentiation of gingival mesenchymal stem/progenitor cells (G-MSCs) [16, 17]. Thus, in the current study AA was incorporated in the PRF in the above concentration and introduced for sustained-release into the intra-osseous defects, to exploit these cellular reparative/regenerative attribute-boosting effects on the resident periodontal stem/progenitor as well as differentiated cells during the surgical wound healing.

The stepwise linear regression analysis demonstrated that irrespective of the treatment group, RLDD at baseline and FMBS after 6 months were significant predictors of CAL gain. This underlines the importance of an inflammation-free periodontium during the healing phase for an enhanced CAL gain. In the present investigation, patients were instructed into regular tooth brushing, and professional plaque control was conducted monthly during the study period, to ensure a plaque- and inflammation-free periodontium. Both OFD+AA/PRF and OFD+PRF demonstrated statistically significant CAL gain and PPD reduction at 6 months, in dimensions comparable to a previous study on EMD+PRF in the treatment of intra-osseous defects [26]. Yet, gingival recession depth reduction and radiographic intra-osseous depth fill were significantly superior in the OFD+AA/PRF group. These findings could be explained by the additive role of AA to the PRF, boosting cellular pluripotency, proliferative and regenerative attributes of stem/progenitor cells, osteoblasts, fibroblasts [69, 70] and G-MSCs [16, 17]; its ability to increase extracellular matrix production [71]; the collagen biosynthesis of the periodontal ligament, gingiva, cement and alveolar bone [72, 73]; and the expression of alkaline phosphatase and osteocalcin [74, 75] as well as its potential to increase the proliferation of keratinocytes and fibroblasts, improving thereby the gingival phenotype [76, 77].

Still, the present results should be interpreted in light of the current trials’ limitations. First, although a 6-month follow-up was employed in previous clinical trials on PRF, limiting the current study’s follow-up to 6 months was greatly attributed to the fact that patients form lower socio-economic status, who usually visit the outpatient clinic of the Faculty of Dentistry, Cairo University for symptomatic treatment, are not interested to adhere to longer follow-up periods. Second, the preparation and use of a blood-derived product as PRF depends on the patients’ acceptance, and hence patients who were afraid of blood sampling refused to participate in the study. Third, the strict inclusion criteria of stage-III grade C periodontitis patients lengthened the duration for participants’ inclusion. Fourth, the current study did not employ the recently developed horizontal centrifugation/preparation protocol, which could have increased the number of platelets and leucocytes in the PRF plugs, with a more even platelets distribution [78, 79], thereby further improving the PRF’s reparative/regenerative effects. Fifth, although the defects were randomized, more favourable defect morphologies (number of osseous walls and defect angel) were arbitrarily allocated to the control than the test group at baseline, a factor that could have affected the outcome of the interventions [42, 80]. Finally, no microbiological or biomarkers examinations were carried out, to test the effect of the interventions on the resident periodontal flora.

Within the limitations of the current randomized controlled clinical trial, it can be concluded that both interventions showed significant improvements in clinical and radiographic outcomes 6 months post-surgically. Augmenting PRF with AA resulted in further significant improvement in gingival recession and radiographic defect fill. The results spot the light on a positive impact of AA and PRF in the treatment of periodontitis and the possibility of their combined application in clinical periodontal therapy. Further clinical and histological studies with longer follow-ups and larger sample size are needed to explore their periodontal regenerative potential.

## Supplementary information

Supplemental figure S1: Consort flow diagram for patients’ recruitment. (DOC 52 kb)

ESM 1 (DOC 219 kb)
