# Supplementary material for: Clinical and radiographic effects of ascorbic acid-augmented platelet-rich fibrin versus platelet-rich fibrin alone in intra-osseous defects of stage-III periodontitis patients: a randomized controlled clinical trial
Source: Clin Oral Investig. 2021 Apr 12;25(11):6309–19. doi: 10.1007/s00784-021-03929-1 (PMC8531044; doi:10.1007/s00784-021-03929-1)
Supplement: Supplementary file 1 — Consort flow diagram for patients’ recruitment. (DOC 52 kb) [file 784_2021_3929_MOESM1_ESM.doc]

**
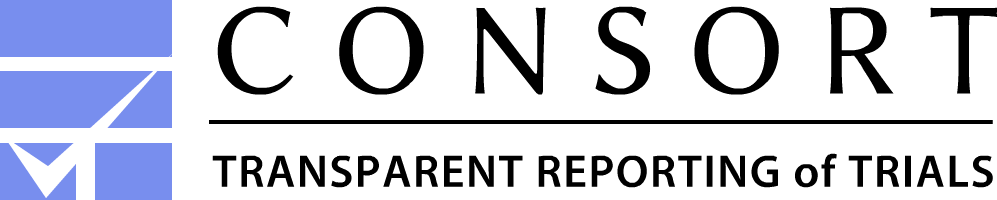
**

**CONSORT 2010 Flow Diagram**

**Allocation**

**Analysis**

**Follow-Up**

**Enrollment**

Assessed for eligibility (n= 43)

Excluded (n= 23)

♦  Not meeting inclusion criteria (n= 20 )

♦  Declined to participate (n= 3)

♦  Other reasons (n= 0)

Analysed (n= 10)
♦ Excluded from analysis (give reasons) (n= 0)

Lost to follow-up (give reasons) (n= 0)

Discontinued intervention (give reasons) (n= 0)

Allocated to intervention (n= 10)

♦ Received allocated intervention (n= 10 )

♦ Did not receive allocated intervention (give reasons) (n= 0 )

Lost to follow-up (give reasons) (n= 0)

Discontinued intervention (give reasons) (n= 0)

Allocated to intervention (n= 10)

♦ Received allocated intervention (n= 10)

♦ Did not receive allocated intervention (give reasons) (n= 0 )

Analysed (n= 10)
♦ Excluded from analysis (give reasons) (n= 0)

Randomized (n= 20)
